# Glioblastoma in the contralateral cerebral hemisphere with previous surgery for meningioma: A case report

**DOI:** 10.1097/MD.0000000000032616

**Published:** 2023-01-06

**Authors:** Zhe Wang, Shushu Sun, Kunming Xie, Junjie Miao

**Affiliations:** a Department of Neurosurgery, Weifang People’s Hospital, Weifang, China; b Department of Education and Training, Weifang People’s Hospital, Weifang, China.

**Keywords:** glioblastoma, meningioma, surgery

## Abstract

Meningioma and glioblastoma (GBM) are 2 common intracranial tumors with different pathophysiologies and prognoses. It is rare for these 2 kinds of tumors to occur in the same patient. Most of the similar cases reported in the literature have been treated with radiotherapy, while cases without radiotherapy are rare. In particular, GBM in the contralateral cerebral hemisphere after resection of meningioma has not been reported.

**Patient concerns:**

We present a case of a 66-years-old man with GBM in the right temporal lobe after previous resection of a benign meningioma of the left frontal lobe without radiotherapy.

**Diagnoses:**

The patient was admitted to our hospital for the first time because of right upper limb weakness. Brain magnetic resonance imaging indicated a space-occupying lesion in the left frontal area. Surgical treatment was performed, and postoperative pathology confirmed a meningioma. The patient was readmitted to the hospital 3 years after surgery of the meningioma due to a new lesion of the right temporal lobe and underwent reoperation. The postoperative pathological results showed GBM.

**Interventions:**

The patient underwent 2 operations, and the postoperative pathologies were meningioma and GBM. In addition, the patient received concurrent chemoradiotherapy and 2 cycles of temozolomide adjuvant chemotherapy.

**Outcomes:**

During the last 4 months of follow-up, the patient was in good condition with no recurrence of the tumor.

**Lessons:**

The development of GBM without radiotherapy after meningioma surgery is very rare, especially at different sites, and it is necessary to accumulate relevant cases to reveal the causes of the disease and provide more evidence for the treatment of similar patients in the future.

## 1. Introduction

Glioblastoma (GBM) is a common intracranial malignant tumor with an annual incidence of approximately 3.1 per 1,00,000.^[1,2]^ Despite standardized comprehensive treatment, including surgery, radiotherapy, and chemotherapy, its prognosis is still poor, and the 5-year survival rate is less than 10%.^[3]^ Meningiomas are different from GBM, with most being benign tumors that are treated with surgery and radiation therapy and have a good prognosis. Only a small number of meningiomas have a poor prognosis, including anaplastic meningiomas and meningiosarcoma.^[4]^ It is very rare for these 2 kinds of tumors with completely different pathological types to occur in the same patient, and most of these patients have a history of radiation therapy.^[5]^ Moreover, several patients with meningioma and GBM at the same time without a history of radiotherapy have also been reported.^[6,7]^ However, new-onset GBM is even rarer after meningioma surgery without radiation therapy. Nazarov VV and Sahuc P et al reported 2 cases of patients with GBM in the same anatomical position as the previous meningioma resection.^[8,9]^ In contrast to these patients, in the case we report herein, a new GBM developed in the contralateral cerebral hemisphere 3 years after meningioma resection. A review of the literature reveals that this is the first case of its type to be reported.

## 2. Case presentation

**Figure 1.**
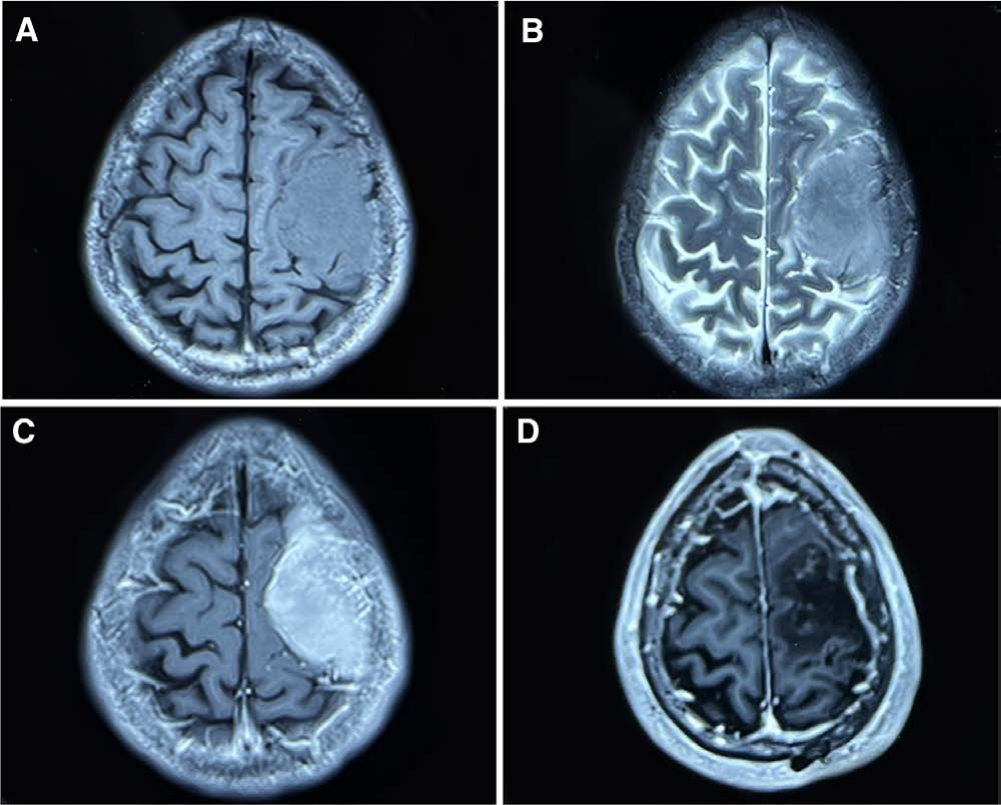
Preoperative MRI scans of the meningioma: *T*1-weighted, *T*2-weighted and *T*1-weighted with contrast enhancement (a, b and c, 7/18/2019); Postoperative MRI scans of the meningioma: *T*1-weighted with contrast enhancement (d, 12/26/2019), MRI = magnetic resonance imaging.

**Figure 2.**
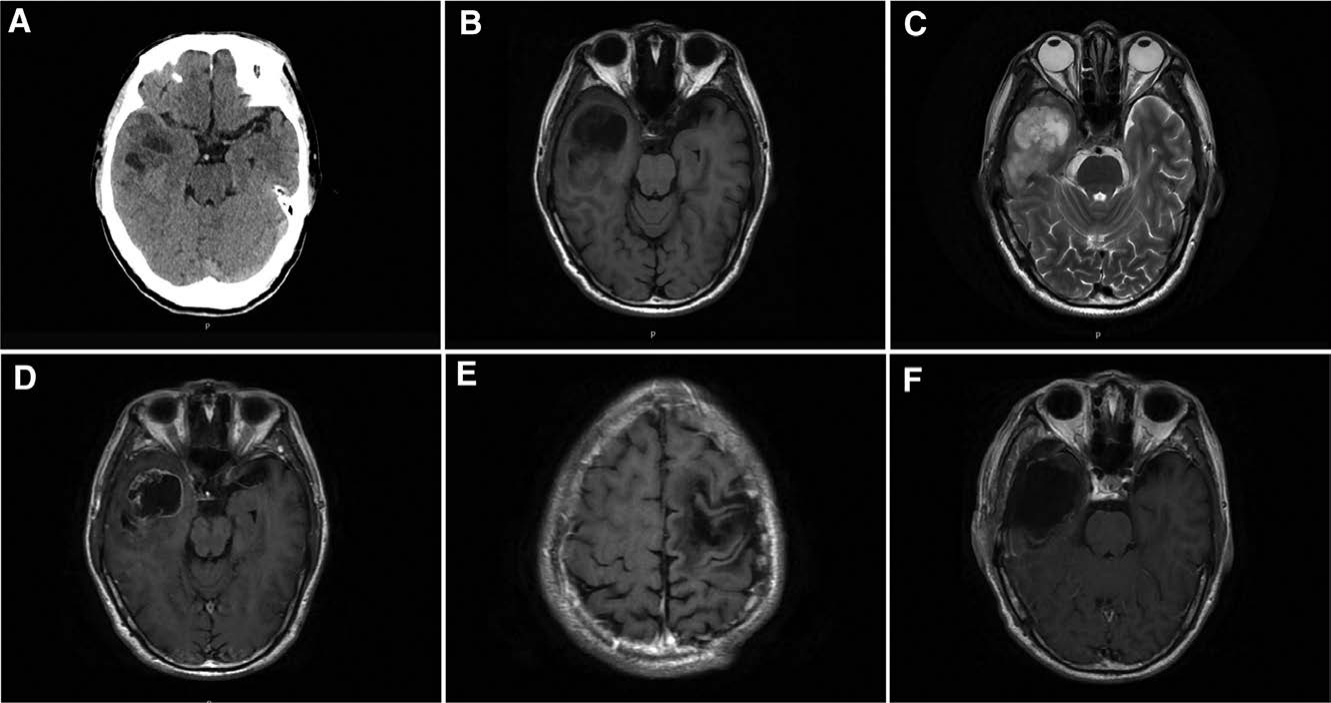
Preoperative CT scans of the GBM (a,7/13/2022); Preoperative MRI scans of the GBM: T1-weighted, T2-weighted and T1-weighted with contrast enhancement (b, c and d, 7/8/2022); Preoperative MRI scans of the meningioma location showed no recurrence: T1-weighted with contrast enhancement (e,7/8/2022); Postoperative MRI scans of the GBM: T1-weighted with contrast enhancement (f, 7/17/2022), CT = computerized tomography, GBM = glioblastoma, MRI = magnetic resonance imaging.

**Figure 3.**
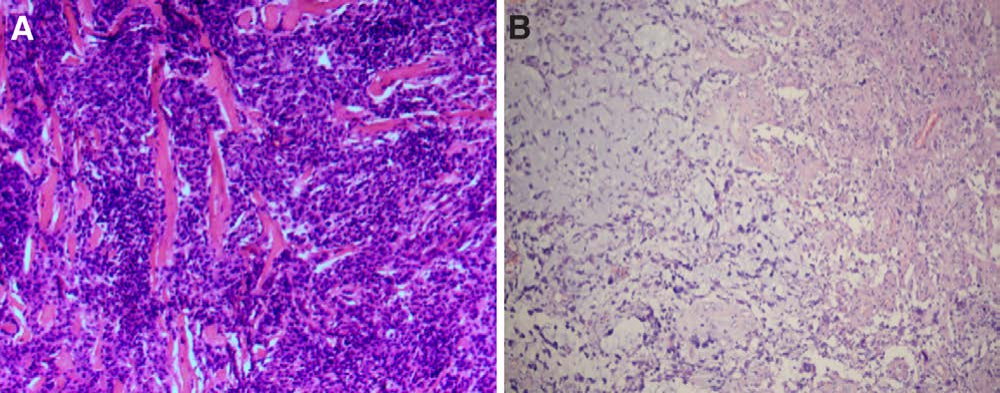
Pathology of the meningioma (a, H&E stain, 7/25/2019); Pathology of the GBM (b, H&E stain, 7/20/2022), GBM = glioblastoma.

A 66-years-old male patient with right upper limb weakness for 1 month presented to our hospital on July 16, 2019. He had no other medical history or genetic disorders. Physical examination after admission found decreased muscle strength of the right upper extremity, with no obvious abnormalities on the rest of the physical exam. A cerebral computerized tomography scan showed a slightly higher density semicircular lesion under the left frontal bone with local cranial hyperostosis. Magnetic resonance imaging (MRI) revealed a lesion of approximately 4.3 × 6.3 × 3.0 cm with high *T*2 and iso-intense *T*1 signals in the left frontoparietal region. The adjacent brain parenchyma was compressed, and there was a cerebrospinal fluid gap between the lesion and the adjacent brain parenchyma. The MRI enhancement sequence showed significant homogeneous enhancement of the tumor and the adjacent dura (Fig. 1a, b, c). After admission, the patient underwent frontoparietal approach craniotomy and achieved gross total resection of Simpson grade I (Fig. 1d), and the postoperative pathology was mixed meningioma (WHO grade I) (Fig. 3a). Since the meningioma was completely excised and proven to be Simpson grade 1, the patient did not receive postoperative radiotherapy. No tumor recurrence was found on MRI at regular follow-up. Incredibly, the MRI scan of this year’s follow-up showed that a new occupying lesion was located in the right temporal lobe with a size of 6.0 × 3.7 × 3.1 cm, while there were no obvious signs of recurrence of the original meningioma in the left frontal area (Fig. 2e). The lesion was cyst-solid and had mixed signals in the solid part, heterogeneous ring enhancement and no obvious interface with the surrounding edematous cerebral parenchyma (Fig. 2a, b, c, d). Magnetic resonance spectroscopy demonstrated that the choline peak was increased, the N-acetyl aspartate peak was significantly decreased, and the choline/ N-acetyl aspartate ratio was significantly increased in the right temporal lobe lesions. Based on the computerized tomography and MRI radiographic findings, we first considered the possibility of high-grade glioma. The patient underwent reoperation on July 15, 2022, during which the cystic wall was cut first to release the yellow–green cyst fluid, and then the solid part of the tumor underwent a piecemeal resection (Fig. 2f). The tumor parenchyma was grayish red with abundant blood supply and no obvious boundary with the surrounding brain tissue. Postoperative pathology confirmed the preoperative diagnosis of GBM (Fig. 3b). The patient recovered well after surgery without complications and accepted concurrent chemoradiotherapy and 2 subsequent cycles of adjuvant chemotherapy with temozolomide. At the last 4 months of follow-up, the patient was in good condition with no recurrence of the tumor.

## 3. Discussion

Patients suffering from meningioma and GBM at the same time are extremely rare. Most of the cases reported thus far have a history of adjuvant radiotherapy or have systemic diseases, such as neurofibromatosis or Von Hippel–Lindau disease.^[10]^ The reason for the recurrence of GBM in these patients may be related to radiotherapy because radiation is 1 of the high-risk factors for glioma. However, some researchers have reported that patients with meningioma without previous radiotherapy developed GBM in adjacent sites. The cause of its occurrence is controversial. Some researchers speculated that meningioma changes the surrounding environment and leads to glioma transformation, especially to the glial cells surrounding the meningioma, which may be the origin of gliomas. For the occurrence of GBM in the same anatomical location as the original meningioma after surgery, Moorthy RK et al supposed that meningioma surgery is similar to traumatic brain injury and may cause local inflammatory reactions and thereby induce the occurrence of glioma.^[11]^ However, many authors do not agree with this hypothesis because glioma rarely occurs in patients with craniocerebral trauma and surgery. In addition, some researchers suggested that the occurrence of 2 tumors at the same time is accidental and that there is no definite correlation between the 2 tumors because they are among the most common tumors in the brain.^[12]^ Moreover, Pauline Sahuc suggested that the occurrence of this condition may be related to the presence of some of the same signaling pathway abnormalities in both gliomas and meningiomas^[9,13,14]^; however, the specific molecular mechanisms need to be further studied in these patients. Interestingly, in our case, and in contrast to similar cases previously reported, GBM did not occur in the same location but in the contralateral hemisphere after primary meningioma surgery without any systemic diseases or radiotherapy. The exact cause of this phenomenon is puzzling. Due to the lack of reports on such patients, further studies of similar cases are needed to uncover the specific mechanism of its pathogenesis.

In conclusion, it is extremely rare for patients without a history of radiotherapy to develop both meningioma and GBM, especially GBM in the contralateral cerebral hemisphere after previous meningioma surgery. To date, the cause of its pathogenesis is not clear. This case report is helpful to the goal of accumulating similar cases, in the hope of uncovering its pathogenesis in the future.

## Author contributions

**Conceptualization:** Junjie Miao.

**Data curation:** Junjie Miao.

**Funding acquisition:** Zhe Wang.

**Project administration:** Zhe Wang.

**Software:** Kunming Xie.

**Writing – original draft:** Junjie Miao.

**Writing – review & editing:** Shushu Sun.
